# An Italian Online Survey Regarding the Use of Hyaluronidase in Previously Hyaluronic Acid-Injected Noses Looking for Surgical Rhinoplasty

**DOI:** 10.1093/asjof/ojac060

**Published:** 2022-07-04

**Authors:** Samuel Staglianò, Gianpaolo Tartaro, Dario Bertossi, Michele Pascali, Valerio Finocchi, Nicola Zerbinati, Pierfrancesco Bove, Pierfrancesco Cirillo, Romolo Fragola, Raffaele Rauso

**Affiliations:** Multidisciplinary Department of Medical-Surgical and Dental Specialties, Oral and Maxillofacial Surgery Unit, University of Campania “Luigi Vanvitelli”, Naples, Italy; Multidisciplinary Department of Medical-Surgical and Dental Specialties, Oral and Maxillofacial Surgery Unit, University of Campania “Luigi Vanvitelli”, Naples, Italy; Maxillofacial Surgery Department, Department of Oral and Maxillofacial Surgery, University of Verona, Verona, Italy; Dermatology Department, University of Insubria, Varese, Italy; Multidisciplinary Department of Medical-Surgical and Dental Specialties, Oral and Maxillofacial Surgery Unit, University of Campania “Luigi Vanvitelli”, Naples, Italy; Multidisciplinary Department of Medical-Surgical and Dental Specialties, Oral and Maxillofacial Surgery Unit, University of Campania “Luigi Vanvitelli”, Naples, Italy

## Abstract

**Background:**

Nonsurgical nasal reshaping (nSNR) with hyaluronic acid (HA) filler is a well-established procedure performed to ameliorate nasal appearance and is considered a valid alternative to surgical rhinoplasty in selected patients.

**Objectives:**

The aim of our study is to evaluate the decision-making process and management of patients undergoing rhinoplasty, with previous HA filler injection, and evaluate if consensus could be achieved to recommend guidelines.

**Methods:**

Between April and May 2021, an online survey was sent to 402 Italian surgeons of different specialties. The survey collected information regarding the types of treatment of patients who have previously undergone nSNR, who should undergo surgical rhinoplasty. For those surgeons using hyaluronidase, an additional information was collected.

**Results:**

In a range of time of 2 months (April and May 2021), a total of 72 surgeons replied and completed the survey: out of the 402 questionnaires sent, the response rate was approximately 18%. The majority of respondents (61.5%) replied to inject hyaluronidase (HYAL) in patients who had to undergo a rhinoplasty but reported previous nSNR. Of the surgeons who use HYAL, 70% performed rhinoplasty after a waiting time of 3 to 4 weeks.

**Conclusions:**

Either direct surgical approach or hyaluronidase injection first seems to be a viable options. The use of HYAL before surgery is the choice with the broadest consensus in our survey. However, a larger case-control study with long follow-ups is necessary to understand if in patient seeking surgical rhinoplasty who already received nSNR, the injection of hyaluronidase before surgery is mandatory, recommended, or not.

Nowadays, nonsurgical nose reshaping (nSNR) with hyaluronic acid (HA) fillers is a worldwide renowned procedure.^1^ This medical procedure has gained popularity because it can be performed in an office setting, does not requires days off for the patient, and can improve nasal deformities such as tip drooping, low to medium prominent nasal dorsum, and small postsurgical deformities.^2-10^ Another favorable aspect of HA in correcting nasal deformities is its long-lasting results.^7^

HA fillers, when introduced into the market in 1998, gained popularity over permanent substances due to HA’s behavior and also for its intrinsic transitory effect, represented by a progressive resorption induced by endogenous hyaluronidases naturally present in the dermal layer.^11,12^ However, when injected into the nose, HA has shown really long-lasting results with a high degree of patient satisfaction.^12^

Some studies have shown long-lasting clinical effects following deep nasal injection of HA: a theory proposed by Mashiko et al stated that deep HA injections, above/onto the periosteum, stimulate periosteal stem cells.^13^ On the other hand, previously injected HA into the soft tissue of the nose (subcutaneous fat/nasalis superficial musculoaponeurotic system [SMAS]), even several months/years earlier, has shown to be still not resorbed at the time of surgery.^10,14^

Nonsurgical nose reshaping, also known as rhinofiller, can be managed with several substances, resorbable and not resorbable; among the others, HA is preferred due to the possibility to be dissolved with hyaluronidase (HYAL) injections.^15^ The first medical report regarding nSNR with HA was published around 2006,^4^ although this procedure has evolved over time;^5-10^ HA drops injected for nasal reshaping can be considered, and used, as “cartilage grafts” that are routinely applied during surgical rhinoplasties.^5^

Several studies have shown that the nose is a challenging area to be injected due to its high vascular network. In fact, vascular compression or embolization following nasal filling can induce skin necrosis, visual loss, or impairment. HA is the most used filler due to the possibility to reverse the vascular impairment with HYAL injection, even if performed several days after vascular impairment, and several papers have shown its effectiveness.^1,16,17^

On the other hand, rhinofiller with HA can also work as a preview for patients seeking surgical rhinoplasty but not sure about the result to achieve; Ramos et al have shown that after one or more nasal injections patients can opt for a definitive surgical correction of their noses.^18,19^ Due to the long-lasting clinical effect of nasal HA injections, the surgeon who has to perform a rhinoplasty in a previously injected nose can have the doubt about whether to inject HYAL or not. At the moment, there is a lack of consensus about this topic.^20-22^

The aim of our study is to evaluate the decision-making process and management of patients undergoing rhinoplasty, with previous HA filler injection, and evaluate if consensus could be achieved to recommend guidelines. In addition, we want to inform future rhinoplasty practice about the correct steps to follow in this particular but increasingly frequent surgical procedure.

## METHODS

Between April and May 2021, an online survey was sent to 402 Italian surgeons of different specialties (Plastic Surgeons; Ear, Nose, and Throat [ENT]; and Maxillo-Facial Surgeons; Appendix). The majority of surgeons participating in the study were Italian Association of Aesthetic Plastic Surgery (AICPE) members (96%); the remaining 4% were reached out directly by the senior author (R.R.) and were enrolled due to their renowned activity mainly focused on nose surgery. The first question was related to the years of practice in surgical rhinoplasty; the second one was about the number of rhinoplasties performed per year. Question 3 was related to whether HYAL injection was performed or not in cases of previously injected noses with HA that had to be operated on. For those who were used to injecting HYAL in previously injected noses with HA, another question was related to the time frame between HYAL injection and surgery.

Only those surgeons who replied “C” on question 2 were included in the study. On the other hand, those who replied “A” or “B” were not considered eligible. A parallel analysis was performed to assess differences between the group of surgeons included in the study and the group of non-eligible surgeons. IRB approval was not necessary for this study.

## RESULTS

In a range of time of 2 months (April and May 2021), a total of 72 surgeons, 56 AICPE members, and other 16 surgeons previously enrolled by the senior author (R.R.) because of their high experience in nose surgery replied and completed the survey: out of the 402 questionnaires sent, the response rate was of approximately 18% (72 surgeons).

**Figure 1.**
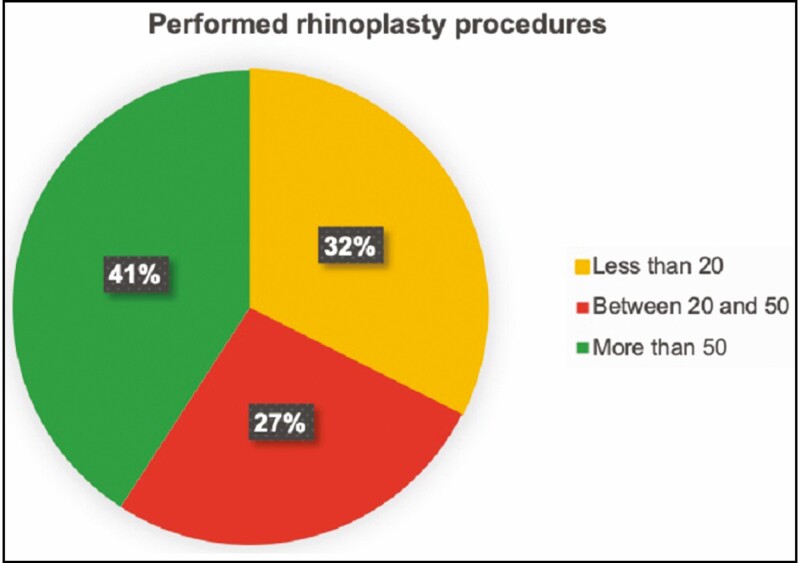
Performed rhinoplasty procedure.

Only 41.6% (30 surgeons) of the participants interviewed were eligible according to our criteria, as they reported performing more than 50 surgical rhinoplasties per year (Figure 1). Among these surgeons, 76.7% have been performing rhinoplasty procedures for more than 10 years (23 surgeons), 20% for more than 5 years but less than 10 years (6 surgeons), and 3.3% for less than 5 years (1 surgeon) (Table 1).

**Table 1.** Practice in Rhinoplasty

Practice in rhinoplasty	Number	Percentage (%)
Less than 5 years	1	3.3
More than 10 years	23	76.7
More than 5 years but less than 10 years	6	20

**Figure 2.**
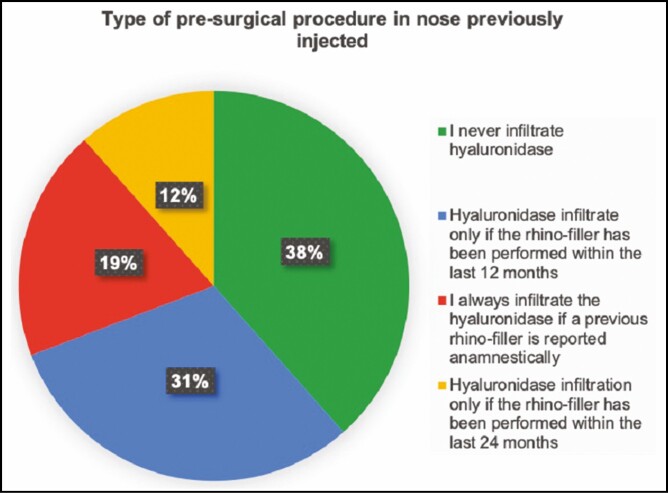
Type of presurgical approach in nose previously injected with hyaluronidase.

**Figure 3.**
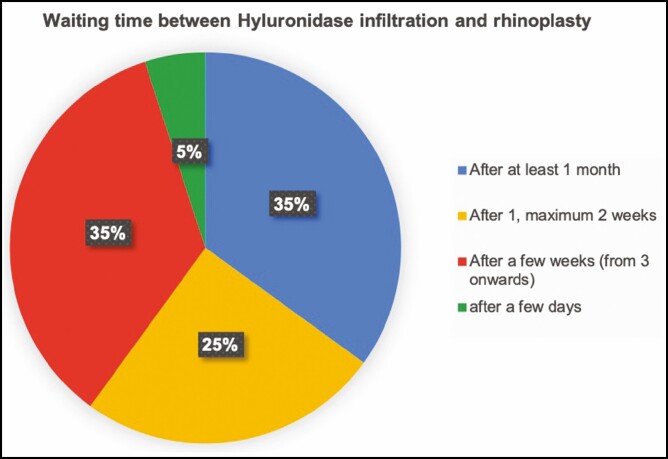
Waiting time between hyaluronidase infiltration and rhinoplasty.

In question 3, surgeons were asked if they performed HYAL injections in patients who had to undergo a rhinoplasty but reported previous HA fillers; 38.5% replied to never inject HYAL before the surgical procedure. The remaining 61.5% replied to inject HYAL: 30.8% always infiltrate hyaluronidase if nasal HA injections were anamnestically reported; 19.2% replied to inject HYAL only if nSNR was performed in the previous 12 months; 11.5% inject HYAL only if nSNR was performed over the last 24 months (Figure 2). The fourth question, regarding how to behave in case of HYAL injection before surgical rhinoplasty: 35% replied waiting at least 1 month, 35% wait a time lapse between 3 and 4 weeks, 25% wait 1 to 2 weeks, and only 5% wait a few days (Figure 3). In our parallel analysis, discordance between the eligible and ineligible groups was evident (Table 2).

**Table 2.** Difference Between Eligible and Ineligible Surgeons

Type of surgeon	Use of HYAL	Time between HYAL and nose surgery
Eligible surgeons	61.5%	3/4 weeks (70%)
Ineligible surgeons	Only 30% (nonuse of HYAL is higher in older surgeons and those who perform few rhinoplasties per year)	3/4 weeks (80%)

HYAL, hyaluronidase.

## DISCUSSION

The nose is considered a keystone in facial aesthetic balance, and until the beginning of the last century, the only treatment option was the surgical approach. The first report about nonsurgical nose reshaping with injectables dates back to the middle of the 1980s, although in that time HA did not exist and collagen and silicone were used for this purpose.^23,24^ Since its introduction, HA has superseded the others due to its excellent safety profile, molding capability, and temporary nature.^12,14,25^ HA fillers were approved by the FDA for the first time only in 2003.^26^ HA fillers are used on the face for several purposes; based on their rheology, they can be used to improve fine lines, get volume enhancement, bony projections, etc.; moreover, HA fillers developed for body contouring also exist.^27^ At the moment, there is no commercially available filler specifically formulated for nasal filling. HA nasal injections were firstly introduced in 2006 in order to ameliorate small irregularities following surgical rhinoplasty with the aim of avoiding secondary surgery.^28^ However, over the years, various techniques have been developed to reach a nonsurgical nose reshaping, thus expanding the indications of HA injections and making nSNR an actual alternative to a surgical treatment.^5,29,30^ Nowadays, nasal remodeling with HA fillers seems to be preferred to surgery due to the absence of days off needed, avoidance of anesthesia, the possibility to perform it in an office setting, and for economic reasons: nSNR is in fact more economic compared with surgical rhinoplasty and it is socially more in vogue.^25,31^ Over the past 15 years, the number of articles dealing with nSNR has steadily increased, showing long-lasting results after injections on both the superficial, epi-periosteal, and peri-chondral planes.^19,28^ In several reports, it is highlighted that only in a small percentage of patients, the clinical result has vanished within 12 months of the injections, showing that injected HA remains longer in immobile structures, such as the nose, compared with other facial areas.^5,6,10,13,14,25,32,33^ The duration of HA further increases when it is injected to correct postsurgical nasal deformities.^10,34^ Due to the aforementioned issues, as surgical rhinoplasty is one of the five most requested procedures in the world according to ASAPS 2020 (352,555 performed rhinoplasties), it is important to note how often rhinoplasty surgeons have to face an increasing number of patients who require surgical rhinoplasty after receiving nSNR.^18,19^ It is important to point out that previous HA injections do not represent a contraindication to subsequent surgery as previous permanent filler injections may be; however, a surgeon should consider it as a “non-primary” procedure. The presence of residual filler, particularly at the peri-chondral level, could cause difficulties during dissection and may modify, with time, the final result.^34^ Before proceeding with surgery, it is, therefore, necessary to carry out a correct medical history of the patient, a careful evaluation of the previously injected filler, the injected layer, and also consider the possible presence of fibro-scar tissue.^11^ Once the surgeon has carefully evaluated the patient and concluded that surgery is the best option, there are several possible approaches.

In theory, as highlighted in other papers, there are 3 possible choices;^18^ (1) wait, even years, in order for HA filler to be resorbed by the action of the endogenous hyaluronidases; (2) remove or “try to remove” the filler during surgery; and (3) inject exogenous hyaluronidase and then perform surgery. In the first case, waiting, even for years, in order to get filler resorption autonomously, could be questionable due to unpredictable and long-lasting results observed in HA-injected noses. It is not always possible to perform a correct clinical evaluation of the injected nasal structures, and as highlighted by Bektas et al a possible alternative is the use of ultrasound (US) examination before undergoing surgical rhinoplasty to evaluate the nature of the filler used and the possible presence of residual volume, or using the clinical transluminescence examination (Tyndall effect).^10^ HA fillers are resorbable fillers; although when injected into the nasal area, they seem to have unpredictable results, and for this reason, it is not possible to precisely predict when HA will be totally resorbed before performing rhinoplasty surgery. Therefore, given the numerous variables, careful clinical and US examination in the presurgical evaluation is of considerable importance.

Removing or “trying to remove” all the fillers during surgery is another option. Also, in these cases, it has been suggested to perform an US examination in order to detect the position of HA before establishing a correct surgical dissection plane.^18^ However, if a rhinoplasty surgeon decides to approach a previously injected nose directly with surgery, it is important to be prepared for 2 scenarios. If a careful preoperative study has been performed and HA is positioned at the same level as the dissection plane, it will be more easily removed. On the other hand, if HA is positioned on a different plane compared with the dissection one, it will be more difficult to remove, and more exploration of the soft tissues will be required, with a greater risk of inducing scar tissue developing and altering the final result. Furthermore, if HA is positioned too superficially, at the level of the subcutaneous plane, it is impossible to remove it using this surgical approach. An alternative approach was proposed by Ramos et al where, through the creation of a tunnel, the identified HA can be removed using an 18G needle and a syringe.^18^

Thirty-eight percent of the eligible surgeons in our survey declared to perform the surgical procedure without previous HYAL injections. The main questionable aspect of this approach is related to the predictability of the final result: what happens if the HA is not completely removed during surgery? What if during the postoperative period the results worsen due to HA reabsorption or due to excessive scar tissue formation? Several medico-legal issues can be raised regarding this topic.

The third approach consists of the injection of HYAL before surgery in order to degrade the previously injected HA. Lambros and Soparkar et al described the first case of HYAL use in order to reverse HA accumulation.^35,36^ Since then, several studies have been conducted about the use of this enzyme, in order to clearly understand its capability to degrade HA filler, and also to eventually face vascular impending adverse events developed following HA injections such as skin necrosis, thrombotic occlusion, or vascular compression.^37-39^ As shown in Figure 2, the majority of more experienced surgeons in our survey (more than 50 rhinoplasties performed per year) prefer this approach before surgery (62%).

Despite various evidence regarding a greater permanence of the filler at the nasal level,^18,40^ the involved rhinoplasty surgeons have not yet reached an agreement about choosing the timing of HYAL injections (Figure 2). Surgeons who gave positive feedback for HYAL injections in nSNR patients were asked a further question regarding the time lapse between the injection of the enzyme and the surgery. Most of them (70%) replied waiting, on average, between 3 and 4 weeks; however, unified consensus was not observed. Only 5% of the involved surgeons wait just a few days before performing rhinoplasty, 25% for a period between 1 and 2 weeks, 35% for at least 3 weeks, and 35% after 1 month. (Figure 3).

An important element to highlight emerged from a parallel analysis of the sample of surgeons excluded from our survey. Among these, there was a greater tendency not to use the HYAL (70%). This trend is more evident among surgeons who perform less than 20 rhinoplasties per year (30%) and in those with more experience (rhinoplasty surgery for more than 10 years) (70%). Even in this parallel analysis, however, among those who use HYAL, the majority (80%) perform rhinoplasty 3 weeks after the injection of the enzyme.

HYAL acts also on endogenous HA, which plays an important role in wound healing,^41^ so surgeons may wonder whether the use of hyaluronidase can influence postsurgical healing processes and after how long it is safe to perform a surgical rhinoplasty following HYAL injections. Even if medical literature clearly shows that, once injected, HYAL activity lasts about 24 to 48 hours, an immediate surgical approach is not advisable due to the potentially associated intraoperative and/or postoperative problems related to tissue inflammation.^26,42^ By evaluating HYAL range of action and endogenous HA turnover at the level of fibroblasts (5 g/ day), Bektas et al empirically suggested to perform the intervention in a time lapse ranging from 1 week to 6 months following HYAL injection.^10,43^

As stated by the ASAPS in 2020, 55,436 rhinoplasty procedures were performed in the United States. Nevertheless, even though nonsurgical rhinoplasty is among one of the fastest-growing aesthetic procedures worldwide, and surgical rhinoplasty is one of the most performed facial aesthetic procedures, we still lack specific guidelines on how to approach patients already injected with HA who are seeking a surgical rhinoplasty. This paper was written with the aim of proving possible indications, but the low response rate (18%) we faced represents a limitation of the present study.

A recent scoping review of the present topic was released revealing that the approach of patients looking for surgical rhinoplasty who already received nSNR is a topic without scientific evidence. Either direct surgical approach or hyaluronidase injection first seems to be viable options with the total absence of postoperative complications.^44^

## CONCLUSIONS

Given the continuous increase of patients waiting for surgical rhinoplasty after HA infiltration, it is necessary to establish a solid presurgical evaluation protocol for these patients. Despite the different therapeutic options available, based on the results of this survey, the use of HYAL before surgery is the choice with the broadest consensus. However, considering the limitations present in this paper and the low response rate to the questionnaire (18% of surgeons interviewed), a larger case-control study with long follow-ups is necessary to understand if in patient seeking surgical rhinoplasty who already received nSNR, the injection of hyaluronidase before surgery is mandatory, recommended, or not.

## Supplementary Material

ojac060_suppl_Supplementary_Appendix
