# Supplementary material for: An Italian Online Survey Regarding the Use of Hyaluronidase in Previously Hyaluronic Acid-Injected Noses Looking for Surgical Rhinoplasty
Source: Aesthet Surg J Open Forum. 2022 Jul 4;4:ojac060. doi: 10.1093/asjof/ojac060 (PMC9317161; doi:10.1093/asjof/ojac060)
Supplement: ojac060_suppl_Supplementary_Appendix [file ojac060_suppl_supplementary_appendix.docx]

**Appendix** Survey questions

1. How long have you been performing rhinoplasties?

A. Less than 5 years

B. More than 5 but less than 10 years

C. Over 10 years

2. How many rhinoplasties per year do you perform?

A. Less than 20

B. Between 20 and 50

C. More than 50

3. How do you behave if you have to perform a rhinoplasty on a patient who reports anamnestically to have undergone a rhino-filler with hyaluronic acid:

A. Hyaluronidase infiltration only if the rhino-filler has been performed within the last 12 months

B. Hyaluronidase infiltration only if the rhino-filler has been performed within the last 24 months

C. I always infiltrate the hyaluronidase if a previous rhino-filler is reported anamnestically

D. I never infiltrate hyaluronidase

4. In case of hyaluronidase infiltration in the nose, according to your personal approach, after how long do you think you can perform a surgical rhinoplasty:

A. After a few days

B. After 1, maximum 2 weeks

C. After a few weeks (from 3 onwards)

D. After at least 1 month

E. After several months: specify ………………… ..
